# Finishing the odyssey to a stem cell cure for type 1 diabetes

**DOI:** 10.1038/s44324-024-00014-5

**Published:** 2024-07-22

**Authors:** Lise Hunault, Daniel Hesselson

**Affiliations:** Centenary Institute and Faculty of Medicine and Health, The University of Sydney, Sydney, NSW 2006 Australia

**Keywords:** Type 1 diabetes, Metabolic engineering

## Abstract

For over two decades pluripotent stem cells have promised a renewable source of β cells to treat patients with type 1 diabetes. Major efforts to optimize the differentiation, survival, and function of transplanted stem cell-derived tissue have recently delivered clinically meaningful metabolic benefits using a perforated encapsulation device that promotes integration with recipient vasculature under the protection of systemic immunosuppression. Despite this success, the journey is not over as a universal cure will require a larger β cell mass. Here, we summarize recent interdisciplinary advances that could maximize the functional β cell mass within transplanted devices and provide an immune privileged niche that could eliminate the need for systemic immunosuppression.

## Introduction

A recent clinical study by Pipeleers and colleagues has brought the possibility of a stem-cell based cure one step closer^1^. This perspective will summarize the major hurdles that have been overcome to deliver cell-based improvements in glucose control and highlight the key issues that stand between this important proof-of-concept clinical study and a durable cure for the majority of patients living with T1D.

### Rationale for stem cell therapies in T1D

The autoimmune destruction of pancreatic β cells creates a lifelong dependence on insulin to control blood sugar levels in individuals with type 1 diabetes (T1D). Over time, poorly managed T1D causes microvascular and macrovascular complications that significantly impact quality of life^2^. Unfortunately, intensive glucose lowering therapy to reduce these long-term complications of hyperglycemia is accompanied by an increased risk of hypoglycemic events^3^. Technological solutions aiming to replace β cell function with an “artificial pancreas” can improve glucose control by integrating continuous glucose monitoring with automated insulin delivery^4^. However, these systems have not yet matched the exquisite blood glucose control provided by human islets^5^, and T1D patients remain burdened with the ongoing management and expense of a chronic disease.

Therapeutic approaches aimed at restoring a functional β-cell mass could eventually eliminate the need for exogenous insulin. Indeed, transplant of cadaveric islets into immunosuppressed T1D recipients has shown that excellent glucose control can be achieved^6^, while simultaneously reducing hypoglycemic risk^7^. The benefits of islet transplant to individual T1D islet recipients should not be minimized, however, the limited supply of donor tissue constrains the potential impact of this strategy, which is still only available to clinical trial participants in many countries including the USA. In contrast, human pluripotent stem cells (hPSCs)^8,9^ could theoretically be expanded and differentiated to restore a functional β-cell mass in all eligible patients with T1D if they can be shielded from autoimmune attack.

Initially, a major goal was to optimize stem cell differentiation protocols to produce glucose-responsive β cells from hPSCs. The first major success was guided by developmental studies from diverse model organisms^10^, in which step-wise modulation of key developmental signals produced β cells capable of expressing insulin^11^, albeit at low levels and in a largely constitutive manner. Nevertheless, this was a remarkable demonstration that hPSCs have the potential to be used for cell-replacement therapy. Extensive empirical optimization and an appreciation of the functional importance of islet structure led to β-cells with improved function^12,13^. We note, the in vitro generation and characterization of stem-cell derived islets has been recently reviewed^14^. However, the observation that in vitro differentiated hPSC-derived β-cells exhibit immature physiological responses^15^, like many other hPSC-derived cell products^16^, led to consideration of alternative strategies. A surprisingly effective approach has involved halting in vitro differentiation once pancreatic fate is established at the multipotent pancreatic progenitor (PP) stage and allowing β-cell differentiation and functional maturation to be guided by endogenous cues post-transplant^17^. An added benefit of this approach is that PP differentiation is amenable to the large-scale expansion and Good Manufacturing Practice (GMP) production and quality control required for clinical application^18^. Interestingly, further differentiation and enrichment of hormone-positive islet-like cells prior to transplant does not reduce the in vivo maturation time^19^.

### Improving encapsulation strategies to protect stem cell grafts

Now that a suitable cell-source is available, preventing graft rejection is one of the greatest challenges facing hPSC-based therapies. The autoimmune nature of T1D poses a challenge for cell-based therapies since the immune system is poised to destroy newly transplanted material, even if it is derived from the patient’s own stem cells. As seen with cadaveric transplants, systemic immunosuppression can protect and maintain unmatched donor β cells in a functional state^6^. Furthermore, clinical transplants have shown that ~10,000 islet equivalents/kg provide a functional β-cell mass that can eliminate the need for exogenous insulin^20^, setting a clear goal for therapeutic effect. Unfortunately, this blunt force approach trades dependence on insulin for continuous immunosuppression, which brings increased risks of infections, certain cancers and regimen-specific toxicities^21^.

Encapsulating transplants in biocompatible materials that prevent immune infiltration, while permitting sufficient diffusion of nutrients and waste products to support β-cell health, has been pursued to eliminate the need for systemic immunosuppression. Despite the demonstration over 40 years ago that microencapsulation is sufficient to preserve islet function for several weeks in an animal model without immune suppression^22^, maintaining a functional β-cell mass within cell-impermeable materials remains a major challenge. Microencapsulated islets (single islets or small clusters) can disperse into the recipient tissue where they benefit from a large contact area with the host. However, the impermeable barrier prevents direct contact with blood vessels, which produce a basement membrane that is likely essential for optimal β-cell function^23^. These problems have been even more pronounced in cell-impermeable macroencapsulation devices, where elaborate designs such as intravascular hollow fibers are used to increase exposure to the bloodstream^24^. However, despite the theoretical advantages of close contact with the blood stream, the serious risk of blood clots associated with vascular prostheses has impeded clinical translation of intravascular devices^25^. The strengths and weaknesses of additional islet encapsulation technologies have been recently reviewed^26^.

**Fig. 1:**
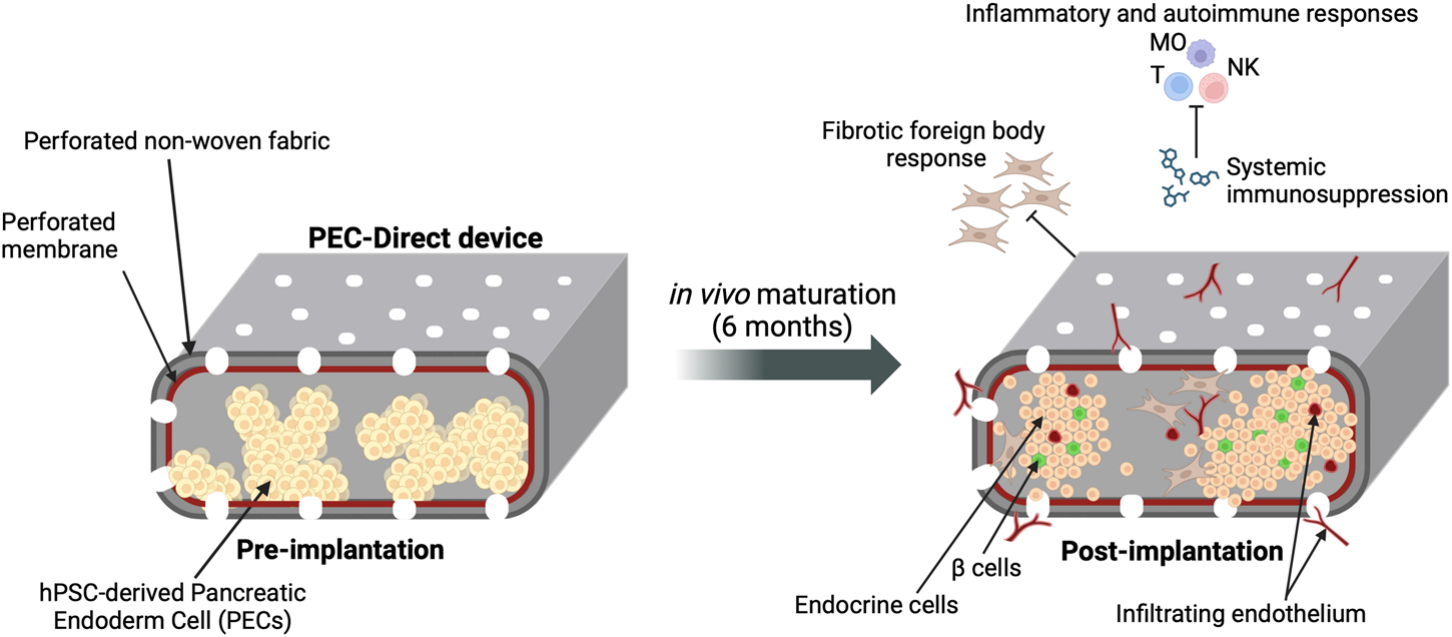
Key features of the clinically successful PEC-Direct encapsulation device. Partially differentiated hPSC-derived PECs were loaded into devices that mature under the protection of systemic immunosuppression in T1D patients. The perforated design facilitates the infiltration of endothelial cells, while the external non-woven fabric restricts fibrotic foreign body responses. After maturation, functional β cells comprised 3% of the total cell mass. MO macrophage, T T cell, NK Natural Killer cell.

Because cell impermeable materials necessarily prevent direct contact between β-cells and the endothelium, some groups have gone a different direction with cell-permeable devices, including Viacyte with the VC-02 device. Although the exact configuration of the VC-02 remains proprietary, key features that appear to have contributed to clinical success are a perforated encapsulation membrane that is encased in another layer of perforated non-woven fabric^27^. The VC-02 device loaded with hPSC-derived Pancreatic Endoderm Cells (PECs) that are partially differentiated to the PP stage has been coined the PEC-Direct (Fig. 1).

### Previous clinical experience with the PEC-Direct device

While most clinical experience is associated with transplant of cadaveric islets to the portal vein in the liver, additional subcutaneous, omental, and intramuscular sites have been extensively studied in preclinical models^28^. These sites may pose additional challenges for islet survival since the limited clinical data available suggests that unencapsulated extrahepatic transplants do not perform well^29^. However, encapsulated hPSC-derived PPs transplanted subcutaneously differentiate into tissue that contains functional glucose responsive β cells within 4-6 months in animal models^30,31^. Building on this experience, two parallel first-in-human studies aimed to optimize the cell dose and perforation configuration of PEC-Direct subcutaneous implants in small numbers of T1D recipients (*n* = 17^32^; *n* = 15^33^) demonstrated that C-peptide, a marker produced by insulin-secreting cells, could be newly detected in some individuals at 6 months post-transplant and could persist until 24 months. A subset of patients achieved >30 pM C-peptide after meal stimulation (6/24, note some individuals were analyzed in both studies), a level that is associated with reduced T1D complications^34^. However, none of the individuals reached the 200 pM threshold associated with improved metabolic control^34^ or the 1000 pM level associated with insulin independence in cadaveric islet recipients^35^. For reference, postprandial C-peptide levels range from 1000-3000 pM in healthy individuals^36^. Importantly, the observed insulin production could be directly attributed to the VC-02 devices, and not the recovery of the recipient’s own β cell function, since removal of the explants eliminated the improvements in C-peptide levels in two patients where this was carefully explored^33^. While comparison of transplanted PEC cells with cadaveric islets in terms of islet equivalents can only be approximated, these pilots delivered at most one-half the transplant volume required for insulin independence. Since the recovered devices contained mostly glucagon+ α cells (16%) and only a small fraction of insulin+ β cells (3%) it is not surprising that the transplants were not sufficient to improve secondary measures of glycemia. Regardless, these first-in-human studies demonstrated the overall safety of the approach in high risk (hypoglycemia unaware) patients with all serious adverse events attributed to the immunosuppressive regimen or surgical procedure, suggesting that maximizing transplant size and β-cell composition were going to be crucial for clinical impact.

### Optimized PEC-direct grafts improve glycemic control in some T1D recipients

In an interim report of 1-year outcomes, Keymeulen *et al*., now provide evidence that hPSC transplants are on the cusp of providing benefit to many patients. Using an adaptive trial design, the transplant volume was increased 2-3 fold and all devices used the perforation pattern and density associated with the best outcomes in previous trials^32,33^ The transplant recipients were selected using similar criteria to the previous trials, requiring stable T1D ( > 5 years), a high risk for hypoglycemic complications (Clarke score ≥4), and meal-stimulated C-peptide levels ≤30 pM prior to transplant. With the increased dose and optimized device configuration, 3/10 recipients produced ≥100 pM postprandial C-peptide from 6-months post-transplant and one surpassed the 200 pM threshold associated with metabolic significance. Excitingly, this individual achieved improved time spent in the target blood sugar range (by continuous glucose monitoring), a clinically meaningful measure of function.

Now that hPSC-derived β cells have been shown to produce metabolically significant amounts of insulin in a T1D patient, there is a path to match and potentially exceed the outcomes observed with cadaveric transplants. Assuming a linear relationship between β-cell mass and insulin secretion, it appears that a further ~10-fold increase in functional β-cell mass would be sufficient to achieve insulin-independence ( > 1000 pM C-peptide) in some patients and a metabolic benefit ( > 200 pM C-peptide) in most recipients. Unfortunately, simply further increasing the transplant size would likely increase surgical complications. Consistent across the clinical trials, recovered PEC-Direct devices contained large acellular regions filled with extracellular matrix. This material permanently occupies space that could be better utilized as β cells currently comprise at most ~3% of the total volume within a device^1^. Although histological analysis of the PEC-Direct devices retrieved from the non-responders was not available in the interim report, further insight into the fate of transplanted PPs and the composition of infiltrating cells in failed grafts will help focus future efforts. Interestingly, in samples from two responders, the less functional graft was already dominated by infiltrating recipient cells at 3 months post-transplant and the β cell mass was negligible at 9 months despite having a larger total cell volume^1^. Human islets are composed of ~50% β cells that are interspersed with other endocrine cell types and aligned to the vasculature^37^. Thus, if the majority of the device volume were filled with islet-like structures, there should be a sufficient functional β cell mass for most patients.

### Pathways to a durable cure for T1D

**Fig. 2:**
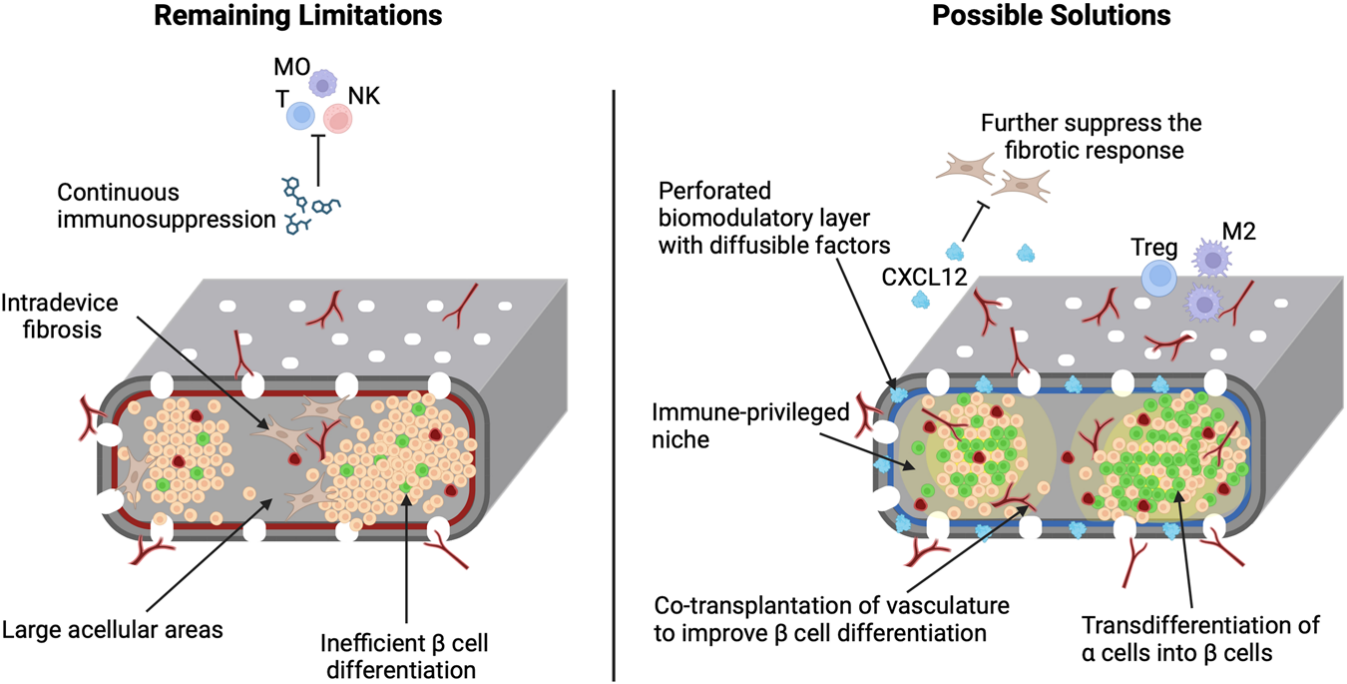
Strategies to increase the functional β cell mass and eliminate systemic immunosuppression. Immunomodulatory materials and cells could be used to create an immune privileged niche for transplanted PECs and further discourage fibroblast infiltration. β cell numbers could potentially be increased by improving the microenvironment and converting other pancreatic cell types to the β cell fate. MO macrophage, T T cell, NK Natural Killer cell. Treg Regulatory T cell, M2 M2 macrophage, CXCL12 CXCL12 chemokine.

Recapitulating embryonic pancreatic development in vitro has produced PPs that clearly have the potential to complete differentiation into functional β cells in a process that takes 4-6 months post-transplant. Additional clues from developmental biology indicate that there are stage-specific interactions between endogenous endocrine precursors and the vasculature that influence pancreatic differentiation. Initially, endothelial cells induce the differentiation of endocrine cells^38^, which then signal back to increase the density of the local vascular network^39^ and deposition of a vascular basement membrane that promotes β cell function^23^. Thus, β cells participate in the construction of a specialized niche through interactions with the vasculature that are essential for subsequent β cell maturation. While the perforated design of the PEC-Direct device allows infiltration of endothelial cells, the growth of this vascular network takes time and is competing with recipient fibroblasts which are only partially blocked by the outer non-woven fabric layer (Fig. 1), suggesting that there are limitations to mechanical control of these processes. The strengths and weaknesses of the PEC-Direct device compared to other cell-based therapies are summarized in Table 1. Here, we highlight recent advances that could help maximize the yield of vascularized β cells and in the best-case scenario provide an immune privileged niche that would eliminate the need for systemic immunosuppression (Fig. 2).

**Table 1:** Strengths and weaknesses of the PEC-Direct device compared to other cell-based therapies

	Advantages	Disadvantages	Alternative approaches in development
Biofouling	∙ Perforated non-woven fabric inhibits the fibrotic foreign body response	∙ Fibroblasts still infiltrate the device	∙ Incorporation of biomodulatory materials^40,41,44^
Vascularization	∙ Infiltrating endothelial cells establish a vasculature network within the device	∙ It takes several months for recipient cell to vascularize the device	∙ Addition of preformed microvessels to enhance vascularization^45,46^
Functional β cell mass	∙ 3/10 recipients produced >100pM C-peptide ∙ 1 /10 transplant achieved metabolically significant function	∙ Insulin+ β cells constitute 3% of the total cell mass ∙ Large acellular areas	∙ Transplantation of enriched populations of fully differentiated beta cells^12,13,19^
Immune tolerance		∙ Requires continuous systemic immunosuppression	∙ Induction of local tolerance with immune cloaked cells^58^

The materials in the PEC-Direct device, particularly the outermost non-woven fabric layer, suppress a full-blown foreign body response associated with the recruitment of macrophages and fibroblasts to the interface with recipient tissues^27^. Limiting residual fibroblast infiltration^1^ might be most important in the acute post-transplant period, as they likely interfere with PP differentiation and the establishment of the intra-device vascular network. The precise composition of the perforated VC-02 encapsulation membrane remains proprietary. However, if it is composed of alginate or similar material, then biomodulatory factors could be directly integrated into the encapsulation membrane^40^. Notably, incorporation of the CXCL12 chemokine was recently shown to protect microencapsulated xenogeneic islets in a non-human primate model^41^. The primary mechanism of acute islet protection is associated with repulsion of islet-reactive effector T cells^42^. However, CXCL12 has multiple immune modulatory roles^43^, and protected islets also show reduced macrophage and fibroblast surface infiltration and collagen deposition^40,41^. These studies suggest that incorporating chemokine(s) such as CXCL12 into the encapsulation membrane, or potentially adding an additional biomodulatory layer, could improve the microenvironment within the device. Additional advances in biomaterials functionalized with diverse immunomodulatory molecules have been recently reviewed in the context of islet transplantation^44^.

Giving the vasculature a head start could be a complementary way to limit the opportunities for intra-device fibrosis. Instead of relying exclusively on the recipients’ vasculature, the addition of ready-made microvessels isolated from adipose tissue to hPSC-derived PPs improved early graft survival and reduced the time required for β cell differentiation to less than 10 weeks in mouse T1D models^45^. Harvesting recipient microvessels would add additional complexity to a clinical transplant program but a proof-concept pilot study using healthy donor microvessels could be informative. Ideally, microvessel-equivalents would also be produced from hPSCs^46^, although scale up under GMP conditions as was done for PPs^18^ would also be needed.

Improving the intradevice microenvironment might increase not only the mature pancreatic cell volume within a device but potentially also the proportion of β cells. In the small number of recovered grafts that have been analyzed histologically, β cells comprise at most ~3% of the total cell volume^1,33,34^. In contrast, preclinical studies with similar device-encapsulated PPs have produced grafts with up to 16% β cells by transplanting into a preformed pouch at the surgical site^47^. Presumably, the 5 weeks between pouch formation and device engraftment allowed for vascularization of the transplant site and resolution of acute inflammatory responses. Importantly, these data indicate that partially differentiated PPs are capable of producing significantly more β cells within an optimized microenvironment. Beyond improving the host environment, an attractive source of additional β cells is from transdifferentiated α cells, which are invariably the most abundant pancreatic cell type identified after in vivo maturation of PPs^1,47^. While paracrine signals from α cells are important for optimal β cell function^48^, these intraislet interactions are unlikely to be compromised by the transdifferentiation of excess α cells that are currently produced in superphysiological proportions. Furthermore, reducing α cell content in the graft could have metabolic benefits as there is growing evidence that hyperglucagonemia interferes with β cell function^49^. α cells have an innate ability to transdifferentiate, although it is only triggered by near complete β cell destruction^50,51^. Overexpression of the key β cell transcription factors PDX1 and MAFA in adult α cells produces β-like cells with the ability to sense glucose and secrete insulin^52,53^, although these cells retain aspects of their previous α cell identity. To avoid perturbing differentiation to the PP stage in vitro, implementing directed transdifferentiation in hPSC-derived transplants will require engineered stem cells with the ability to induce β cell factors specifically in mature endocrine cells^54^. A further 2-3 fold increase of the β cell mass observed in preclinical studies via transdifferentiation would produce structures with very similar cellular composition to endogenous human islets^37^.

The ultimate goal of a hPSC-based therapy for T1D is to provide long-term β cell function without the need for systemic immunosuppression. Cotransplantation of microgels containing individual immunomodulatory factors such as PD-L1^55^ or FasL^56^ can have profound effects on graft survival in immunocompetent hosts. For example, FasL presenting microbeads combined with only two weeks of rapamycin monotherapy supported graft function for over six months and induced Treg-dependent local tolerance without systemic effects on the immune system in an allogeneic mouse model^56^. Similar effects were seen in non-human primates^57^, although the long-term viability of the grafts was not evaluated.

In addition to achieving allogeneic graft tolerance, hPSC-derived β cells must contend with the dysregulated autoimmune response in T1D. Excitingly, it now appears possible to fully cloak hPSCs and their differentiated progeny by overexpressing a cocktail of 8 immunomodulatory factors that includes PD-L1 and FASL^58^. Together, these factors disrupt antigen presentation, T-cell and NK cell attack, and innate inflammatory responses. By activating a proliferation-dependent kill switch^59^, cloaked cells could be maintained in a dormant state within an immunocompetent host. Furthermore, these cloaked cells protected their neighbors, including allogeneic islets and xenogeneic hPSCs. While PPs could potentially be generated directly from cloaked hPSCs, the overexpression of 8 genes might impact β cell function long-term. An elegant strategy to address all the key issues discussed here would be to generate cloaked endothelial cells for cotransplantation with PPs that are genetically primed for α to β cell transdifferentiation.

## Conclusions

Decades of concerted effort have produced the first stem cell derived replacement therapy to show clinical benefit in a T1D recipient. Recent advances in diverse fields illuminate a path to a lifelong cure for this chronic metabolic disease.
